# A retrospective analysis comparing clinical outcome measures pre- and post- the introduction of telehealth in a community-based psychiatry clinic in a tertiary medical centre

**DOI:** 10.1192/bjo.2021.839

**Published:** 2021-06-18

**Authors:** Arup Dhar, Liam Edwards, Moana Waerea

**Affiliations:** 1 Monash Health, Department of Psychiatry, Monash University, Faculty of Medicine, Nursing and Health Sciences; 2 Monash University, Faculty of Medicine, Nursing and Health Sciences; 3 Monash Health, Department of Psychology

## Abstract

**Aims:**

The aim of this retrospective analysis was to look at the effect that telehealth had on patient outcomes and the therapeutic alliance.

**Method:**

Clinical outcomes measures were collected prospectively as part of routine clinical care. Outcome measures were administered at patients’ initial and final appointment. Information was merged into a single database and imported into IBM SPSS for retrospective analysis. The following measures were administered at the beginning and end of treatment and were used to evaluate patient progress; Health of the Nation Outcome Scale (HoNOS), Life Skills Profile (LSP), Session Rating Scale (SRS), Outcome Rating Scale (ORS).

**Result:**

Two cohorts were derived from the clinic; the first cohort (n = 90; 53 females; 37 males; M = 35.72 years; SD = 12.12 years) comprised of those patients whose care occurred between 23/09/2019 and 22/03/2020 and did not receive telehealth appointments. The second cohort (n = 122; 68 females; 54 males; M = 36.2 years; SD = 12.78 years) were those patients who presented to the clinic and were discharged between 23/03/2020 and 21/09/2020 and received at least one telehealth appointment. In the pre-telehealth cohort, mean HoNOS scores at baseline were 17.87 compared to 13.53 at discharge, mean LSP scores at baseline were 10.76 compared to 9.01 at discharge, mean SRS scores at baseline were 34.17 compared to 36.04 at discharge, and mean ORS scores at baseline were 12.97 compared to 21.28 at discharge. In the post-telehealth cohort, mean HoNOS scores at baseline were 14.45 compared to 10.50 at discharge, mean LSP scores at baseline were 7.85 compared to 7.19 at discharge, mean SRS scores at baseline were 36.04 compared to 35.36 at discharge, and mean ORS scores at baseline were 18.83 compared to 15.85 at discharge.

**Conclusion:**

Results show that telehealth did not impact negatively on the therapeutic effect of clinical sessions, highlighted by similar reductions in HoNOS and LSP scores. It was seen in the post-telehealth cohort that there was worsening in the subject-rated scales (SRS and ORS) which was not seen in the pre-telehealth face-to-face cohort. Thus, there seems to be a discernible negative difference from the patient's perspective in the clinical sessions. This may be due to the difficulties in therapeutic alliance using the telehealth platform. We appreciate that there are a number of confounding factors, especially the effect of COVID-19 isolation. Telehealth is a useful addition to our assessment and treatment paradigms and its use should continue; however, we should be aware of the potential negative effect on therapeutic alliance.

